# Copper(II) binding properties of hepcidin

**DOI:** 10.1007/s00775-016-1342-2

**Published:** 2016-02-16

**Authors:** Kanokwan Kulprachakarn, Yu-Lin Chen, Xiaole Kong, Maria C. Arno, Robert C. Hider, Somdet Srichairatanakool, Sukhvinder S. Bansal

**Affiliations:** Faculty of Pharmacy, Payap University, Mae Khao Campus, Chiang Mai, Thailand; Institute of Pharmaceutical Science, King’s College London, Franklin-Wilkins Building, 150 Stamford Street, London, SE1 9NH UK; Department of Biochemistry, Faculty of Medicine, Chiang Mai University, Chiang Mai, 50200 Thailand

**Keywords:** Peptide, Mass spectrometry, Homeostasis, Binding affinity, Biomedicine

## Abstract

Hepcidin is a peptide hormone that regulates the homeostasis of iron metabolism. The N-terminal domain of hepcidin is conserved amongst a range of species and is capable of binding Cu^II^ and Ni^II^ through the amino terminal copper–nickel binding motif (ATCUN). It has been suggested that the binding of copper to hepcidin may have biological relevance. In this study we have investigated the binding of Cu^II^ with model peptides containing the ATCUN motif, fluorescently labelled hepcidin and hepcidin using MALDI-TOF mass spectrometry. As with albumin, it was found that tetrapeptide models of hepcidin possessed a higher affinity for Cu^II^ than that of native hepcidin. The log *K*_1_ value of hepcidin for Cu^II^ was determined as 7.7. Cu^II^ binds to albumin more tightly than hepcidin (log *K*_1_ = 12) and in view of the serum concentration difference of albumin and hepcidin, the bulk of kinetically labile Cu^II^ present in blood will be bound to albumin. It is estimated that the concentration of Cu^II^-hepcidin will be less than one femtomolar in normal serum and thus the binding of copper to hepcidin is unlikely to play a role in iron homeostasis. As with albumin, small tri and tetra peptides are poor models for the metal binding properties of hepcidin.

**Electronic supplementary material:**

The online version of this article (doi:10.1007/s00775-016-1342-2) contains supplementary material, which is available to authorized users.

## Introduction

Hepcidin is a peptide hormone secreted by the liver in response to iron loading and inflammation [1], it controls the export of iron from duodenal epithelial cells, hepatocytes and macrophages and consequently influences the level of serum iron available for the bone marrow and other tissues heavily dependent on iron supply [2–6]. There are more than 200 entries of hepcidin sequences in SwissProt. Some of the common species are shown in Table 1 together with the conservation of the sequence (Table 1).

**Table 1:**
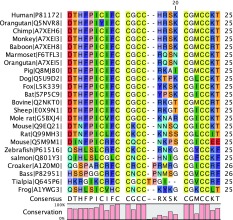
Alignment and conservation of some hepcidin sequences

Interestingly, the N-terminal region of human hepcidin is capable of binding Cu^II^ and Ni^II^ ions via an ATCUN (amino-terminal Cu-Ni)-binding motif comprising the three N-terminal residues (H_2_N-XXH) [7, 8]. In principle almost all hepcidin molecules can chelate Cu^II^ at the N-terminal region, as the majority possess the ATCUN motif, the exceptions amongst the most common sequences, being rat and mouse where position 3 is occupied by asparagine; tialpia, where position 3 is arginine and bass where, although position 3 is serine and position 1 is histidine. Thus even those sequences lacking the ATCUN motif are predicted to bind Cu^II^. Several research groups have demonstrated the binding of transition metals to hepcidin, for instance Farnaud et al. [9] reported the binding of Fe^II^ and Balesaria et al. [10] reported that the regulation of hepcidin levels is influenced not only by Fe^II^, but also Zn^II^, Cd^II^ and Cu^II^. In view of this interest in the divalent metal binding properties of hepcidin, particularly the binding of Cu^II^, we decided to investigate this interaction and to quantify the affinity constant of hepcidin for Cu^II^, such that we could compare the affinity constant with other endogenous Cu^II^ binding proteins and in particular albumin.

**Fig. 1:**
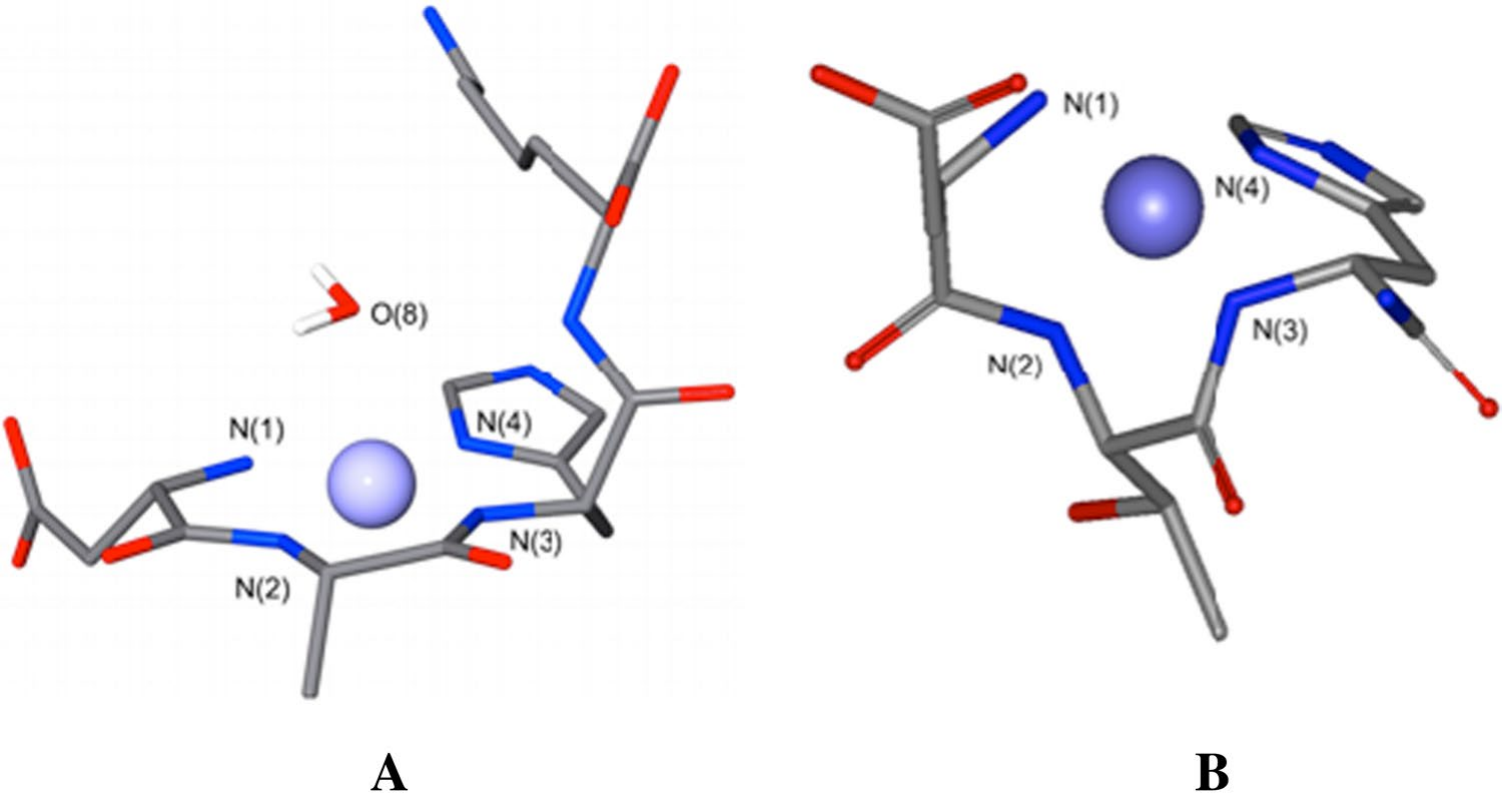
Structure of [Cu^II^(DAHK)] and complex [Cu^II^(DTH)] complexes. **a** X-ray structure of the [Cu^II^(DAHK)] complex. The Cu^II^ ion is penta-coordinated with four nitrogen ligands in equatorial positions (N^1^ to N^4^) and one water molecule in the apical position (O^8^) [12]. **b** Energy minimized structure for [Cu^II^(DTH-NH_2_)] complex

Human serum albumin (HSA), the most abundant protein of blood serum, participates in blood Cu^II^ transport [11]. The N-terminal three amino acid residues for human serum albumin are Asp-Ala-His; the ATCUN motif. On the basis of studies centred on tri- and tetrapeptide mimics of the N-terminus of human albumin it has been suggested that Cu^II^ is bound in a slightly distorted square planar geometry by four nitrogen’s in the equatorial plane: the aspartate amino group, two deprotonated amide functions from the first two peptide bonds and the N^π^ nitrogen of the imidazole side chain of the histidine residue (Fig. 1a) [12].

In principle the structure of the Cu^II^ complex of human hepcidin could adopt a similar structure, as previously suggested by Tselepis et al. [8] (Fig. 1b) who reported the dissociation constant between hepcidin and Cu^II^ as ≪1 µmol/L [8]. As Cu^II^ is a kinetically labile cation [13], both human albumin and human hepcidin might be expected to compete with each other. In view of this possibility it is important to establish an accurate affinity constant for the binding of Cu^II^ to hepcidin. For this reason we have monitored the binding of Cu^II^ to human hepcidin, fluorescent human hepcidin and three peptide models of the N-terminus of human hepcidin, in order to gain a better insight into the possible significance of this potentially important Cu^II^-hepcidin interaction.

## Materials and methods

### Materials

All solutions were prepared with Milli-Q (18 MQ) water. The Fmoc amino acid derivatives were obtained from Bachem AG (4416 Bubendorf, Switzerland). DMF, DMSO, 3-(*N*-morpholino) propanesulfonic acid (MOPS), copper atomic standard solution, acetonitrile, nitrilotriacetic acid (NTA), potassium chloride, Cu(II) sulphate pentahydrate and other reagents were purchased from Sigma-Aldrich and used without further purification. Analytical grade volumetric hydrochloric acid (0.2092 mol/L) and HPLC grade water (Fisher) were used in the preparation of all solutions used for potentiometry.

### Peptide synthesis

In the hepcidin sequence (DTHFPICIFCCGCCHRSKCGMCCKT) Methionine 21 was replaced with a Lysine (M^21^K) which was protected on the ε-amino group with the (4,4-dimethyl-2,6-dioxocyclohex-1-ylidene)ethyl group (Dde). Peptide synthesis was carried out on a CEM Liberty 1 microwave peptide synthesizer with Fmoc-Thr(tBu)-Trt-Resin on a 0.2 mmol scale. All deprotection reactions were carried out with 20 % piperidine in dimethylformamide (v/v). Acylation reactions were carried out using 2-(6-chloro-1-*H*-benzotriazole-1-yl)1,1,3,3-tetramethylaminiumhexafluorophosphate (HCTU) (0.78 mmol) in the presence of 2,4,6-collidine (1.6 mmol) for 10 min using 25 W microwave power. Cysteine and histidine were activated with diisopropylcarbodiimide (0.8 mmol) and ethyl cyano(hydroxylimino) acetate (0.8 mmol). Boc-Asp(OtBu)-OH was incorporated at the N-terminus [14]. Peptides DTH, GGHG and DTHFPIAIF-NH_2_ were prepared similarly on PEG-PS resin.

For the preparation of the labelled hepcidin, the peptide resin (400 mg, 69 μmol) was treated with 15 mL of hydrazine hydrate in dimethylformamide (2 %, v/v) to selectively remove the Dde group. The free amino group was acylated with either dichloro-6-carboxyfluorescein (65 mg, 173 μmol) or 6-carboxy tetramethylrhodamine (45 mg, 104 μmol), diisopropylcarbodiimide (27 μL, 173 μmol) and ethyl cyano(hydroxyimino) acetate (25 mg, 173 μmol) in 2 mL of DMF over 18 h.

The peptide resins were cleaved from the solid support and simultaneously deprotected using trifluoroacetic acid, phenol, water, thioanisole and 3,6-dioxo-1,8-octanedithiol (82.5/5/5/0.5/2.5) for 4 h and precipitated in diethyl ether and then freeze dried.

The linear labelled hepcidin was then dissolved in reducing buffer (100 mL) composed of tris hydrochloride (0.5 mol/L), EDTA (0.1 mmol/L) and guanidine hydrochloride (6 mol/L) at pH 8.0 and reduced with excess dithiothreitol (53 mg, 345 μmol). The fluorescent peptide solution was acidified to pH 2.5 and then purified by preparative HPLC on a Vydac 218TP54 peptide and protein column (2.5 × 30 cm). The folding buffer (6.8 L) was composed of ammonium bicarbonate (100 mmol/L), EDTA (0.1 mmol/L), guanidine hydrochloride (2 mol/L), reduced glutathione (2.10 g, 6.9 mmol) and oxidized glutathione (0.42 g, 0.69 mmol). The purified reduced peptide was dissolved in folding buffer (100 ml) containing 6 mol/L GdmHCl and added to the bulk of the folding buffer. The solution was stirred for 48 h and the solution was purified by preparative HPLC to produce the crude folded peptide.

All peptides were purified by preparative HPLC on a Vydac peptide and protein column 218 TP54 semipreparative column (1.0 × 30 cm) and the resulting fractions were analysed on a Jupiter HPLC column (5 µm, C18, 300 Å, 2 mm × 150 mm, Phenomenex) using a 1100 HPLC system (Agilent Technologies). Mass spectrometry analysis of the peptides was carried out using matrix-assisted laser desorption ionization on a Bruker Autoflex [15]. An Nd:YAG laser (355 nm) was used to irradiate the sample. The instrument was calibrated with a range of peptides covering the mass range (500–4000 Da). Samples were prepared on an Anchor Chip^**®**^ with a saturated solution of α-cyano-4-hydroxy-cinnamic acid as the matrix in a mixture of acetonitrile and 0.1 % trifluoroacetic acid. Direct infusion experiments were carried out on a linear Ion Trap (Thermo). (S^1^,S^8^: S^3^,S^6^: S^2^,S^4^: S^5^,S^7^-tetracyclo-DTHFPICIFCCGCCHRSKCGMCCKT (Hepcidin), S^1^,S^8^: S^3^,S^6^: S^2^,S^4^: S^5^,S^7^-tetracyclo-DTHFPICIFCCGCCHRSKCGK(5-tetramethylrhodamine)CCKT (M12K-TMR-hepcidin) and S^1^,S^8^: S^3^,S^6^: S^2^,S^4^; S^5^,S^7^-tetracyclo-DTHFPICIFCCGCCHRSKCGK(Dichloro-5-carboxyfluoresceine)CCKT (M^21^K-dichloro-CF-Hepcidin) were successfully prepared. Both M^21^K-TMR-hepcidin and M^21^K-dichloro-CF hepcidin exhibited measurable potency when tested in cultured Madin-Darby canine kidney (MDCK) and T47D cell expressing ferroportin [16].

### Potentiometric titration

The potentiometric titration system used in the study of GGHG, GGH and DTH-NH_2_ and their Cu^II^ complexes comprised of an autoburette (Metrohm Dosimat 765 litre mL syringe) and Mettler Toledo MP230 pH meter with Metrohm pH electrode (6.0133.100) and a reference electrode (6.0733.100). A potassium chloride electrolyte solution (100 mmol/L) was used to maintain the ionic strength. The temperature of the test solutions was maintained in a thermostatic jacketed titration vessel at 25 ± 0.1 °C by using a Techne TE-8 J temperature controller. The solution under investigation was stirred vigorously during the experiment. All instruments were interfaced to a computer and controlled by a Visual Basic program. An automated titration adopted the following strategy: the pH of a solution was increased by 0.1 pH unit by the addition of potassium hydroxide solution (100 mmol/L) from the autoburette; when pH readings varied by <0.01 pH unit over a 8 s period, they were judged to be stable and an incubation period was activated. For pKa determinations, an incubation period of 1.5 min was adopted; for metal stability constant determinations, an incubation period of 3 min was adopted. The cycle was repeated automatically until the defined end point pH value was achieved. All the titration data were analysed with the pHab program [17]. The species plot was calculated with the HYSS program [17, 18]. Analytical grade reagent materials were used in the preparation of all solutions.

### Spectrophotometric titration

Following the same procedure as described in 2.3, the spectrum of the solution was additionally recorded at the end of the incubation period. The test solution was circulated through a Hellem quartz flow cuvette by a Gilson Mini-plus #3 pump with speed capability (20 mL/min). The flow cuvette was mounted on an HP 8453 UV–visible spectrophotometer. For pKa determinations, a cuvette path length of 10 mm was used while for metal stability constant determinations, a cuvette path length of 50 mm was used.

### Fluorescence spectroscopy

Fluorescence spectra were obtained using a Perkin Elmer LS50B fluorometer. Excitation and emission frequencies of 552 and 576 nm were adopted, and data were recorded at room temperature. Briefly, sample volumes of 2 ml, with peptide concentrations of 0.5 µmol/L M^21^K-TMR-hepcidin in 0.25 mol/L MOPS buffer (pH 7.45)/acetonitrile (v/v = 50:50) and aqueous 2 µmol/L Cu^II^SO_4_ solution (50 µl) was added every 5 min.

### Mass spectrometry

The peptides were dissolved in a standard copper solution to form a 1:1 peptide to copper preparation. The solution was serially diluted with 20 mmol/L ammonium bicarbonate to prepare peptide—Cu^II^ solutions of different concentrations.

For the preparation of the liquid matrix for MALDI-MS, 10 mg of α-cyano-4-hydroxycinnamic acid, 30 μL of diisopropylethylamine and 50 μL of 10 mmol/L ammonium phosphate in aqueous methanol (50 %) were vortexed and sonicated to obtain a solution. This solution was then diluted 30-fold with 20 mmol/L ammonium bicarbonate: methanol (1:1, v/v). The sample and the matrix (2 μL each) were mixed and then spotted onto a MALDI plate and allowed to evaporate to yield a liquid film. The samples were then analysed by MALDI-TOF.

## Results

In order to gain a better understanding of Cu^II^ binding by the ACTUN motif of hepcidin we have studied Cu^II^ binding to various hepcidin N-terminal peptides. Some are tri- and tetrapeptides and are limited to the ACTUN motif and one DTHFPIAIF-NH_2_, is a nonapeptide representing the N-terminal section of hepcidin with the cysteine being replaced by alanine. We have also studied Cu^II^ binding to human hepcidin and two fluorescently labelled human hepcidin analogues.

### Synthesis of the peptides

The synthesis of the peptides proceeded without any major difficulties the percentage yields of the crude peptides GGHG, DTH-NH_2_ and DTHFPIAIF-NH_2_ were 20, 40 and 60 % respectively. All peptides were greater than 95 % pure as demonstrated by HPLC and corresponded to the correct structures as confirmed by electrospray ionization mass spectrometry (ESI–MS) and/or MALDI-TOF MS data. For the labelled peptides, the linear peptides were successfully synthesised on the solid support by replacing methionine^21^ with Lys(Dde), the Dde group was removed with hydrazine and the free amino group was labelled with either 6-carboxytetramethylrhodamine or 6-carboxy dichloro-carboxyfluoresceine. The peptide was cleaved from the solid support, purified and then folded under oxidative conditions containing oxidised and reduced glutathione to produce the products, which were characterised by HPLC and MALDI-TOF MS (see supplementary data). Both M^21^K-TMR-hepcidin and M^21^K-dichloro-CF hepcidin exhibited measurable potency when tested in cultured Madin-Darby canine kidney (MDCK) and T47D cell expressing ferroportin [16].

### Potentiometric determination of the pKa values of GGHG and DTH-NH_2_

**Fig. 2:**
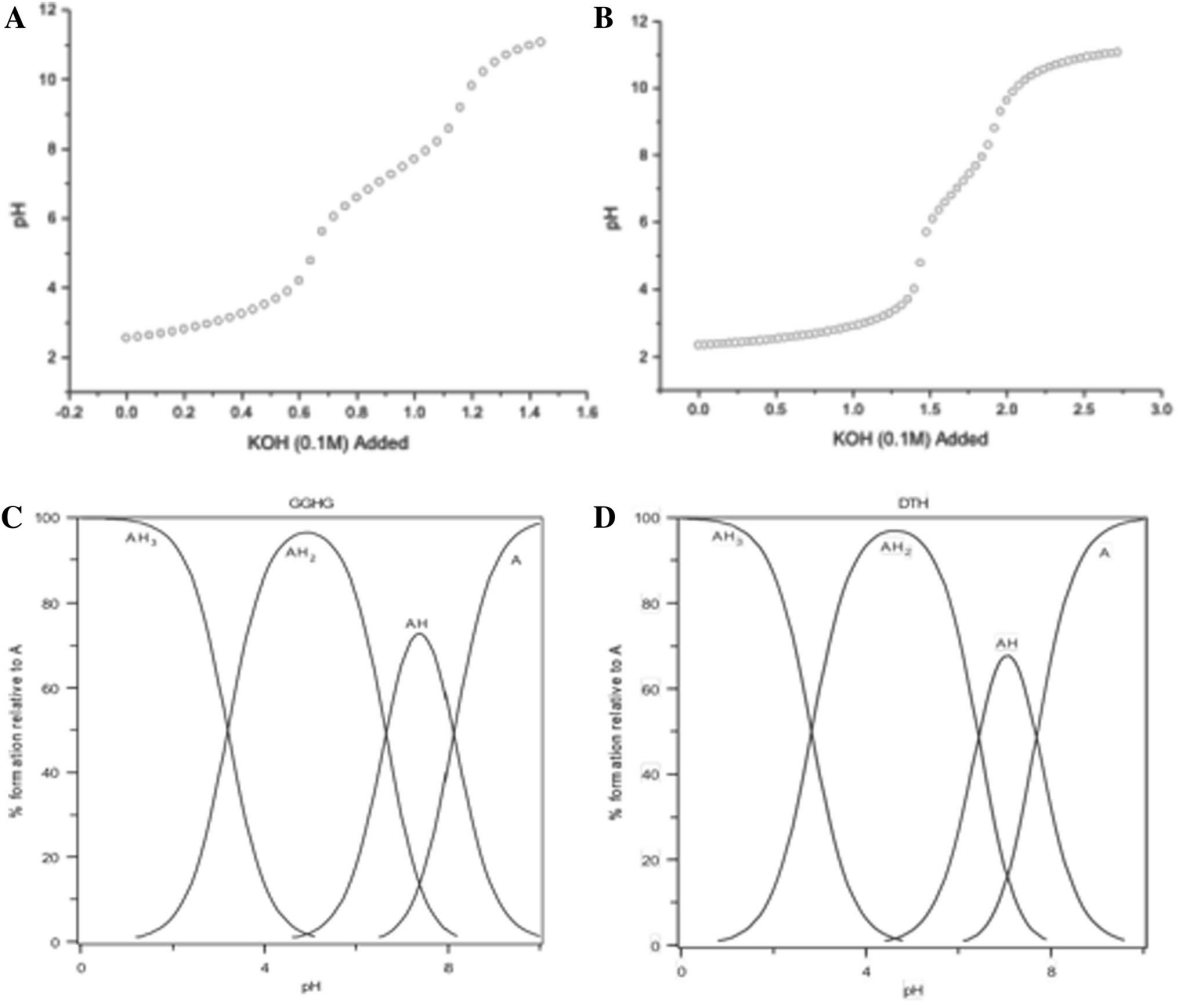
The potentiometric titration of GGHG and DTH-NH_2_. **a** Potentiometric titration of 16.5 mg GGHG with 0.1 M KOH. **b** The speciation plot of GGHG. **c** Potentiometric titration of DTH-NH_2_. 16.6 mg with 0.1 M KOH. **d** The speciation plot of DTH-NH_2_

The potentiometric titration and resulting speciation plot for GGHG are presented in Fig. 2a. The pKa values are 8.1 (terminal amino group), 6.7 (imidazole group) and 3.2 (carboxy function) (Table 2).

**Table 2:** Stability constants of short peptides

Species	GGHG	DTH-NH_2_	GGH
LH	8.1	7.9	8.3
LH_2_	6.7	6.3	6.8
LH_3_	3.2	2.8	2.6
Cu^II^LH	3.7	3.3	2.4
Cu^II^LH_2_	−1.1	−1.4	−1.6
logK_7.4_	12.8	12.9	12.2
pCu_7.4_^II^*	13.8	13.9	13.1

* pCu_7.4_^II^ under condition [ligand]_Total_ = 10^−5^ M, [Cu^II^]_Total_ = 10^−6^ M, pH = 7.4

The predominant form at pH 7.4 is AH where the terminal amino group function remains protonated and the peptide lacks a net charge. The pH titration and resulting speciation plot for DTH-NH_2_ are presented in Fig. 2c. The pKa values are 7.9 (terminal amino group), 6.3 (imidazole group) and 2.8 (carboxy function) (Table 2). The predominant form at pH 7.4 is AH where the terminal amino group function remains protonated and the peptide lacks a net charge.

### Spectrophotometric determination of Cu^II^ affinity constants for GGHG and DTH-NH_2_

**Fig. 3:**
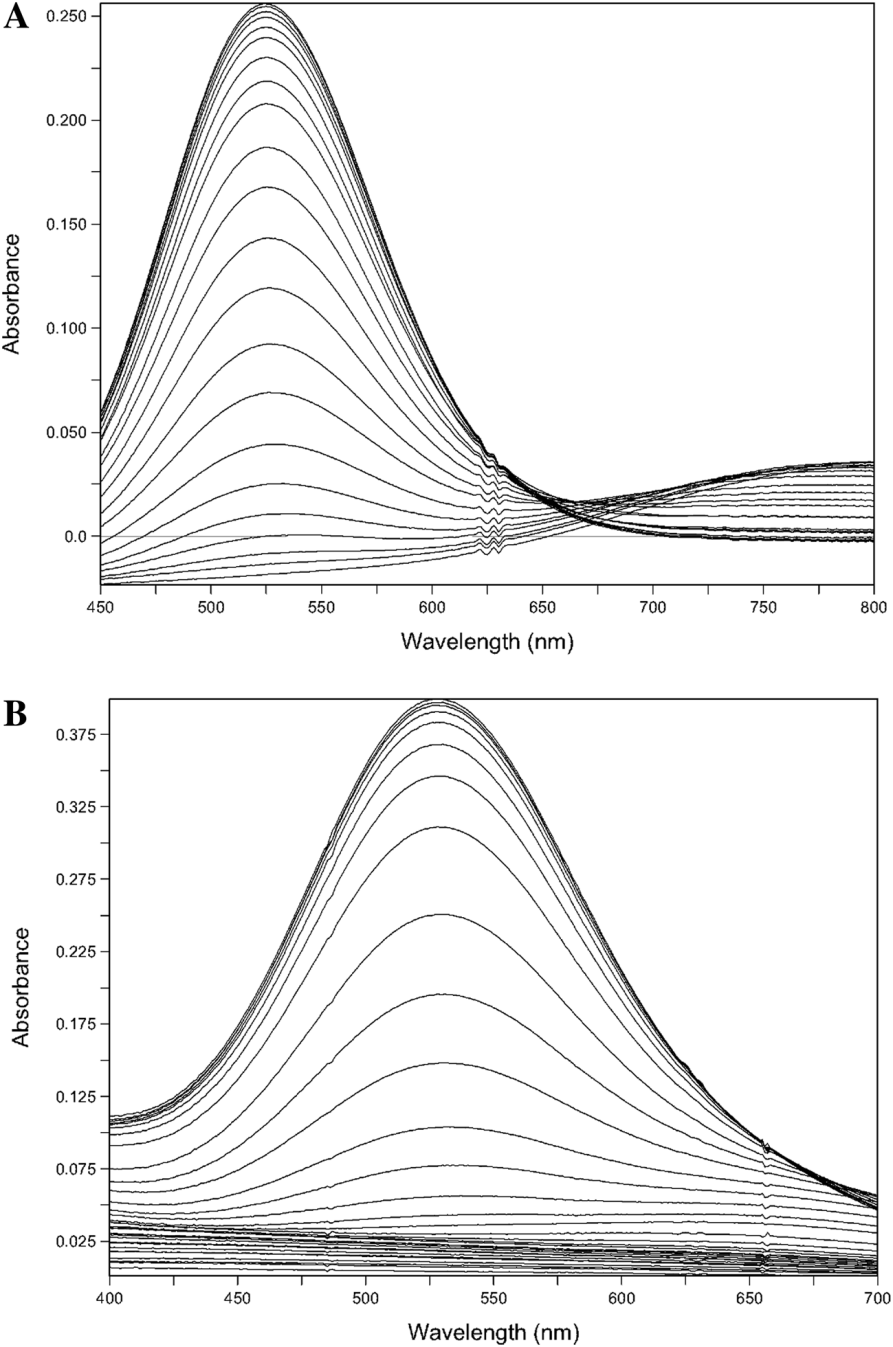
Spectrophotometric titration spectra of GGHG and DTH-NH_2_ with Cu^II^. **a** Titration spectra of GGHG with Cu^II^, [GGHG] = 2.33 mmol/L and [Cu^II^] = 1.11 mmol/L, ratio of L:M = 2.1, starting in 21.741 mL 0.1 M KCl at 25 °C, pH 2.267 to pH 10.030. **b** Titration spectra of DTH-NH_2_ with Cu^II^, [DTH-NH_2_] = 2.94 mmol/L [Cu^II^] = 735.5 µmol/L, ratio of L:M = 4, starting in 20.667 ml 0.1 M KCl at 25 °C, pH 1.814 to pH 9.207

GGHG, spectrophotometric titration in the presence of Cu^II^SO_4_ resulted in the formation of a complex (*λ*_max_ = 528 nm) (Fig. 3a) from which the conditional affinity constant at pH 7.4 was determined, namely log *K*_1_ = 12.84. With DTH-NH_2_, titration with Cu^II^SO_4_ resulted in the formation of a complex (*λ*_max_ = 530 nm) (Fig. 3b) from which the conditional affinity constant at pH 7.4 was determined as log *K*_1_ = 12.93.

These values are close to the reported Cu^II^ log conditional affinity constants for some analogous peptides, that correspond to the N-terminal sequence of human albumin (DAH), for instance DAHK-NH_2_ (13.8) [19] and DAH-NH_2_ (13.7) [20]. It is significant that these two values are higher than the log conditional affinity constant for Cu^II^ and HSA [21].

In order to further investigate the interaction of Cu^II^ with this peptide class we measured the log conditional affinity constants for GGH in order to compare the values determined in this study with the values previously reported for this tripeptide [22, 23] and with the values for GGHG (this study) and GGHGG [22]. The values are all in good agreement with each other despite differing experimental conditions being adopted in the three laboratories (Table 3).

**Table 3:** Cu^II^ Log conditional affinity constants at pH 7.4

Peptide	Log*K*_1_	References
GGH	12.4	[23]
GGH	12.3	[22]
GGH	12.2	This work
GGHG	13.6	[22]
GGHG	13.13	This work

In this investigation, we adopted spectrophotometric titration over the pH range 2.5–10.5, whereas in previous studies, data of the potentiometric titration over the pH range 2.0–7.8 was adopted [22, 23]. With both GGH and GGHG we observed a transition over the pH range 6.5–8.5 which was possibly not recorded by the previous workers. We attribute this transition to the formation of copper complex dimers as previously suggested by Aiba et al. [24].

### Determination of the copper(II) affinity constants for fluorescently labelled hepcidin

**Fig. 4:**
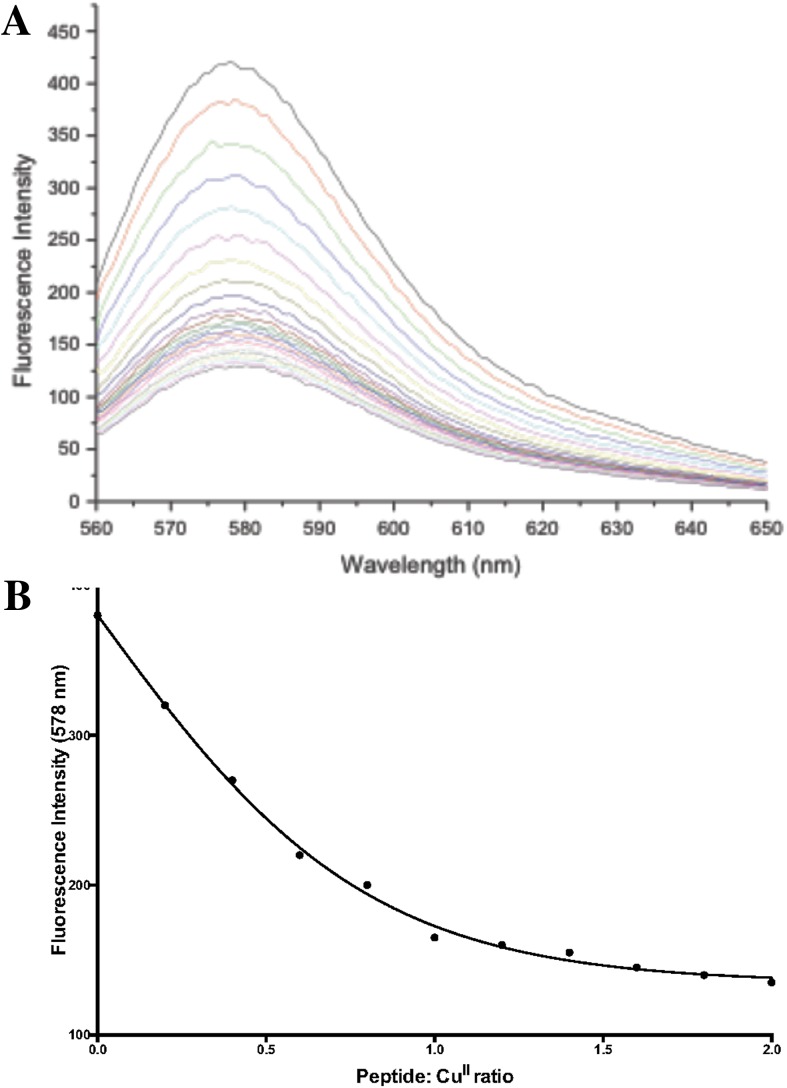
Fluorescence titration of M^21^K-TMR-hepcidin with Cu^II^. **a** M^21^K-TMR-hepcidin (0.5 µmol/L) in MOPS (0.25 mol/L, pH 7.45): acetonitrile (50:50) was quenched by the addition of Cu^II^SO_4_ (2 µmol/L) in 50 µL aliquots; **b** Analysis of the same titration, monitoring the fluorescence at 578 nm

We selected two fluorescently labelled hepcidins which were both derivitised on position-21, a residue believed to be orientated away from the ferroportin binding region of hepcidin. This is somewhat confirmed by both these molecules possessing biological activity [16]. Unfortunately both the labelled hepcidin molecules possess a low solubility in water, although they are soluble in aqueous acetonitrile mixtures. Initially a Cu^II^ titration was undertaken with M^21^K-TMR-hepcidin in 50 % aqueous acetonitrile. Copper was found to quench the fluorescence in a dose dependent manner (Fig. 4). This titration yielded a log conditional affinity constant of M^21^K-TMR-hepcidin for Cu^II^ (log *K*_1_) of 7.5. The quenching studies were repeated at 20, 40, 60 and 70 % aqueous acetonitrile, which rendered it possible to extrapolate the resulting log*K*_1_ values for Cu^II^ to aqueous solution (see supplementary data). The extrapolated value was determined as log*K*_1_ = 7.22. Thus there was acceptable agreement between the two determined Cu^II^ affinity constants, but both were found to be much lower than the affinity constants determined for the N-terminal segments of hepcidin, namely log*K*_1_ in the range 12.2–13.6. It is significant to note that there is a similar difference between the affinities of Cu^II^ of short N-terminal peptides of albumin and albumin itself [19, 21].

The difference in this behaviour is likely to be related to the relative ease of the formation of a square planar Cu^II^ structure, involving the dissociation of two amide protons. Small restrictions in the geometry could lead to relatively large changes in the affinity constant. By analogy with albumin, it is likely that the higher value of Log *K*_1_ (Cu^II^) for the N-terminal peptides, when compared with fluorescently labelled hepcidins, results from an interaction of the ATCUN motif with the rest of the hepcidin structure. Indeed the C-terminal region of hepcidin is close to the N-terminus region in the native structure it being held in such a position by the numerous disulphide bonds present in the structure [6].

### Determination of hepcidin copper(II) affinity constants by mass spectroscopy

Although the two fluorescently labelled hepcidin molecules are likely to interact with Cu^II^ in a similar fashion to that of the native molecule, the possibility exists that the fluorescent probe could interfere with the conformation of the N-terminus region. Consequently we studied the binding of Cu^II^ to a N-terminal model peptide of hepcidin (DTHFPIAIF-NH_2_) and human hepcidin by MALDI-TOF. This is a useful technique for the analysis of biological molecules but has two limitations. Firstly the crystallisation of the matrix and the sample on the plates results in a non homogeneous surface which leads to loss of resolution and secondly most sample preparations are carried out in an acidic environment where metal and/or protein complexes tend to dissociate. In view of these limitations we have used a liquid matrix approach [25] in order to perform the study at physiological pH values and to maintain the homogeneity of the sample. Furthermore as signal suppression is common in MALDI analysis, we omitted the use of an internal standard and the first measurement was carried out using the peptide with no added copper.

**Fig. 5:**
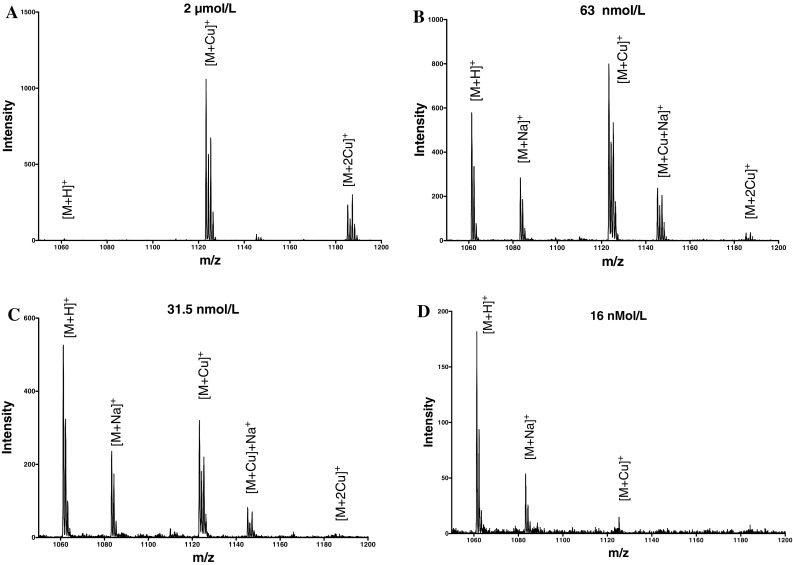
Analysis of DTHFPIAIF-NH2-Cu^II^ complex by mass spectrometry. DTHFPIAIF-NH_2_ and Cu^II^ are present in equimolar amounts. **a** 128 μmol/L; **b** 63 nmol/L; **c** 31.5 nmol/L and **d** 16 nmol/L

By preparing a 1:1 molar ratio of peptide and Cu^II^ at pH 7.0, it is possible to monitor the dissociation of Cu^II^ from the peptide as a function of concentration. As the solution becomes more dilute, Cu^II^ dissociates from the peptide complex (Fig. 5); thus at 2 μmol/L (Fig. 5a) the dominant species is the 1:1 peptide- Cu^II^ complex; at 63 nmol/L (Fig. 5b) the relative size of the free peptide peak increases; at 31 nmol/L (Fig. 5c), the free peptide peak dominates and at 16 nmol/L (Fig. 5d) there is virtually no detectable peptide Cu^II^ complex. From this data we estimate an affinity constant for Cu^II^ and the N-terminal model peptide of 50–60 nmol/L (log *K*_1_ = 8.7–8.8). At the highest copper-peptide concentration (2 μmol/L) there was also a minor contribution in the MS associated with a two Cu^II^/peptide species. Spectrophotometric titration of DTHFPIAIF-NH_2_ with Cu^II^ resulted with an identical affinity constant namely log *K*_1_ = 8.8 (supplementary Figure S7 and Table S1).

**Fig. 6:**
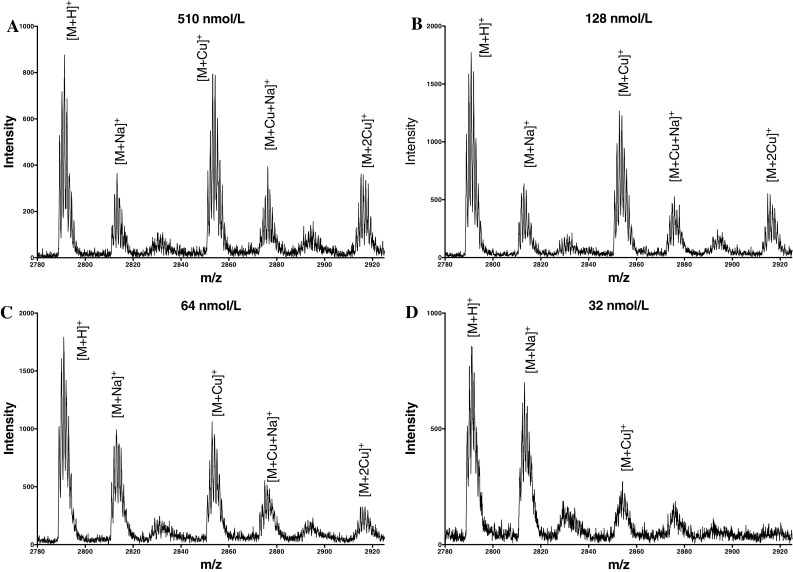
Analysis of hepcidin-Cu^II^ complex by mass spectroscopy. Hepcidin and Cu^II^ are present in equimolar amounts. **a** 510 nmol/L; **b** 128 nmol/L; **c** 64 nmol/L; **d** 32 nmol/L

A similar mass spectra series was obtained with Cu^II^/hepcidin (Fig. 6). At 510 nmol/L the peak heights of hepcidin and the Cu^II^/hepcidin complex are similar (Fig. 6a), at 128 nmol/L the hepcidin peak height is marginally larger (Fig. 6b) and at 32 nmol/L the hepcidin peak height dominates the spectra (Fig. 6d). From these spectra we estimate the Cu^II^ affinity constant of hepcidin to fall in the range 500–600 nmol/L (log *K*_1_ = 7.7–7.8); an order of magnitude lower than that of the N-terminal peptide. As with the N-terminal peptide, a two Cu^II^/hepcidin species was also detected at the higher peptide concentrations. Similar results were also obtained from K^21^(Cl_2_CF)-Hepcidin-Cu^II^ (supplementary Figure S7).

In summary log Cu^II^ affinity constants for the two fluorescently labelled hepcidins were 7.5 and 7.2 and the log Cu^II^ affinity constant for native hepcidin determined by mass spectrometry was 7.7–7.8. These values suggest that the presence of the fluorescent probe only slightly reduces the affinity for Cu^II^. Significantly the log Cu^II^ affinity constant for labelled N-terminal nonapeptide bound to Cu^II^ is approximately tenfold tighter than the hepcidin molecule, again confirming the difficulty of using short peptides to model cations binding to polypeptides.

We conclude that native hepcidin binds Cu^II^ with a log *K*_1_ value of 7.7–7.8. This value agrees with the previous estimate of Tselepis et al. [8] (Log *K*_1_ ≫ 6) and is now sufficiently accurate to undertake speciation studies with potential endogenous Cu^II^ chelators, for instance albumin.

## Discussion

Hepcidin binds Cu^II^ with relatively high affinity (Log *K*_1_ = 7.7), but is this value sufficiently high to compete with intracellular copper chaperone proteins and extracellular proteins, for instance albumin? The intracellular labile copper pool is vanishingly small (<femtomolar) [26] due to the necessity of preventing copper from competing with iron and manganese for enzyme active sites [27]. At such low Cu^II^ concentrations, hepcidin will not be able to bind copper. In contrast the extracellular concentration of labile Cu^II^ is much higher, typically 1–2 μmol/L [28], and the majority of this pool is bound to albumin. The affinity of human serum albumin for Cu^II^ is log*K*_1_ = 12 [21] which is approximately 100,000 times higher than the value for hepcidin as reported in this study. As Cu^II^ is kinetically labile [13], it is expected to rapidly distribute between albumin and hepcidin. The serum albumin concentration is approximately 600 µmol/L, whereas that of hepcidin is of the order of 10 nmol/L. Thus the bulk of kinetically labile Cu^II^, will be bound to albumin. It is estimated that the concentration of hepcidin coordinated to Cu^II^ in normal serum is <1 fmol/L. Thus under both intracellular and extracellular environments, hepcidin will be unable to compete with other endogenous Cu^II^ binding ligands and thus it is unlikely that the binding of copper to hepcidin plays a role in iron homeostasis.

## Electronic supplementary material

Below is the link to the electronic supplementary material.

Supplementary material 1 (PDF 1614 kb)
