# Supplementary material for: Copper(II) binding properties of hepcidin
Source: J Biol Inorg Chem. 2016 Feb 16;21:329–38. doi: 10.1007/s00775-016-1342-2 (PMC4850187; doi:10.1007/s00775-016-1342-2)
Supplement: Supplementary file 1 — Supplementary material 1 (PDF 1614 kb) [file 775_2016_1342_MOESM1_ESM.pdf]

Supplementary Information

Index

1. Fig S1. HPLC chromatograms of GGHG and DTH-NH_2
2. Figure S2 Mass spectra of GGHG and DTH-NH_2
3. Figure S3 HPLC Chromatogram $K^{21}(Cl_2CF)$-Hepcidin
4. Figure S4 MALDI-TOF MS of $K^{21}(Cl_2CF)$-Hepcidin
5. Fig S5. Extrapolation of log conditional Cu^{II} affinities
6 Fig S6 Spectrophotometric titration spectra of DTHFPIAIF-NH_2 with Cu^{II}
7 Table S1 Extrapolation of the Log conditional CuII affinities for DTHFPIAIF-NH_2
8 Figure S7 Analysis of $K^{21}(Cl_2CF)$-Hepcidin -Cu^{II} complex by mass spectroscopy
9. Ferroportin internalisation assay and hepcidin internalisation assay

A

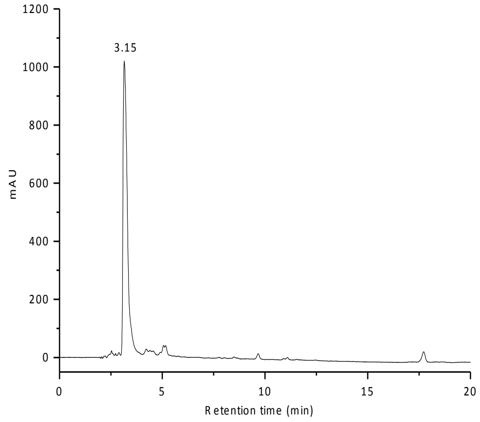

B

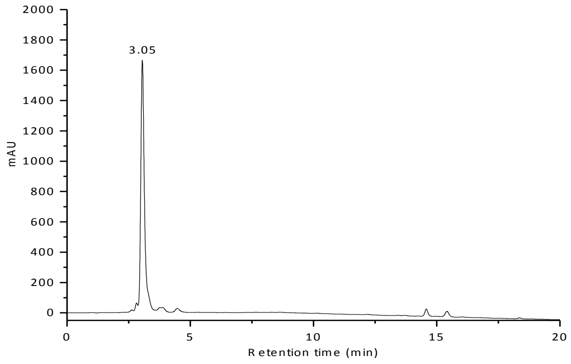

Fig S1. HPLC chromatograms of GGHG and DTH-NH_2
Using a monitoring wavelength of 220 nm, GGHG shows a retention time of 3.15 min. (A) and DTH-NH_2 a retention time of 3.05 min. (B)

A

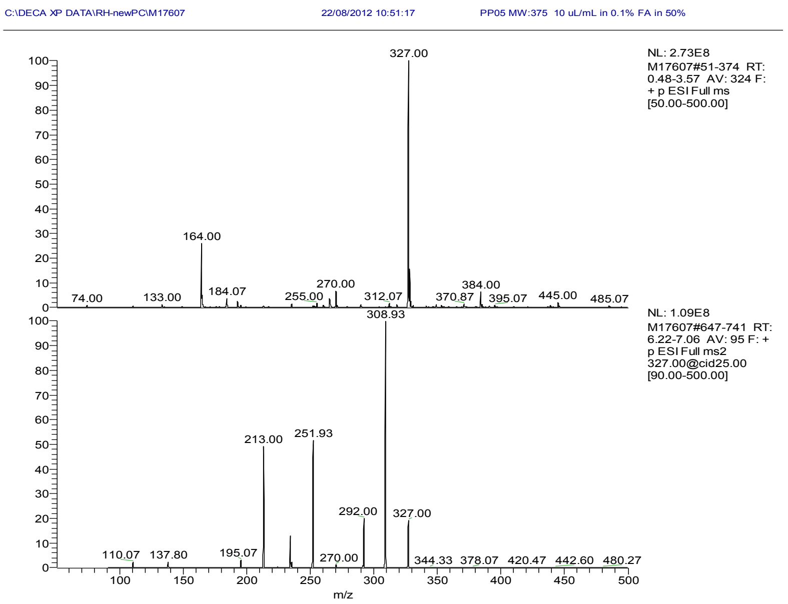

B

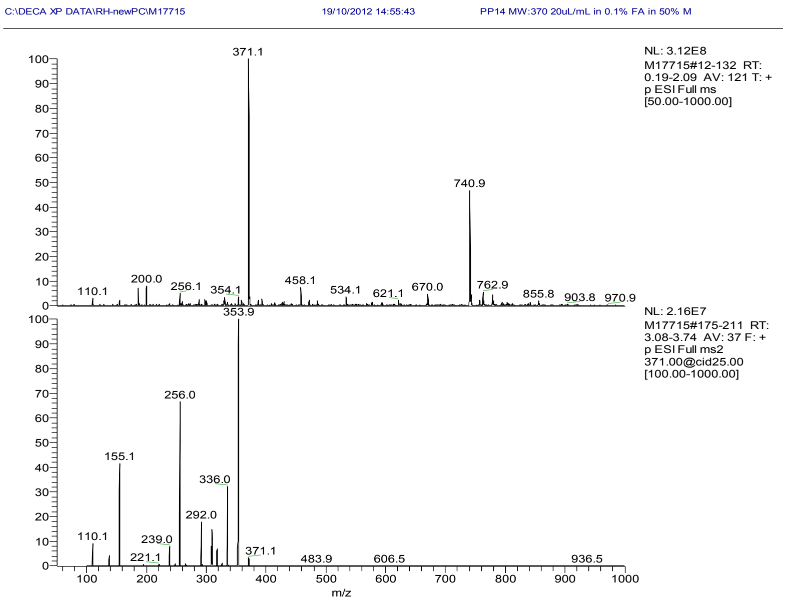

Figure S2 Mass spectra of GGHG and DTH-NH_2

(A) Mass spectrum of GGHG, 10 µl/ 1 ml 0.1% FA in 50% methanol, ESI, MS (-ve) molecular weight of GGHG is 327. (B) Mass spectrum of DTH-NH_2, 20 µl/ 1 ml 0.1% FA in 50% methanol, ESI, MS (-ve) molecular weight of DTH-NH_2 is 371.

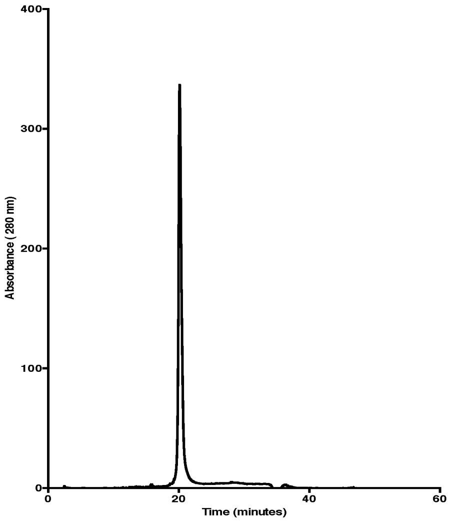

Figure S3 HPLC Chromatogram $K^{21}(Cl_2CF)$-Hepcidin

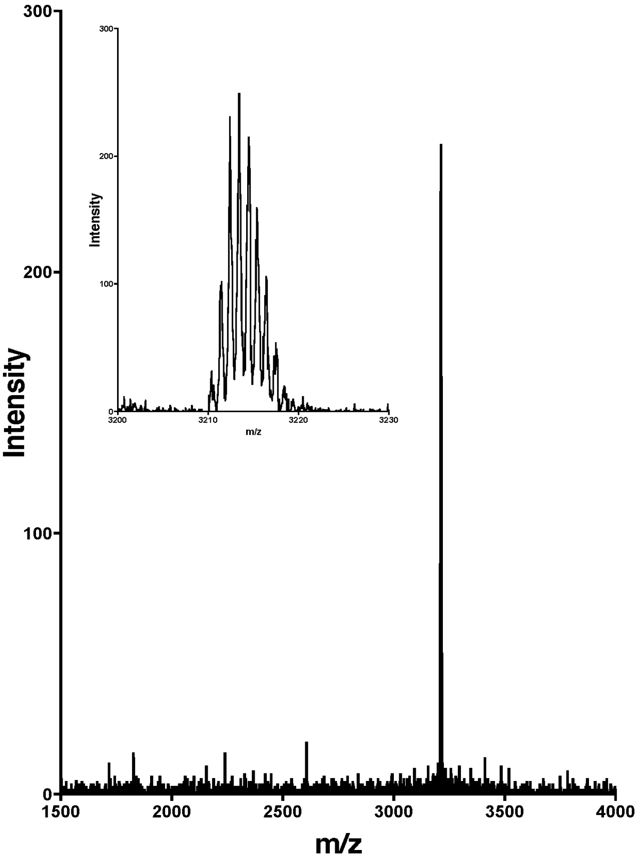

Figure S4 MALDI-TOF MS of $K^{21}(Cl_2CF)$-Hepcidin

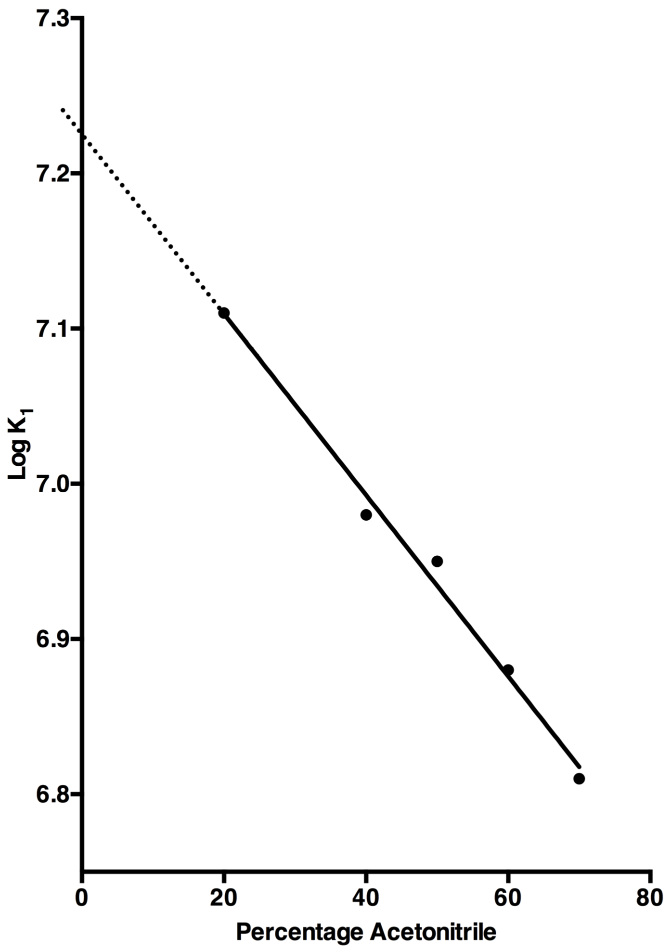

Fig S5. Extrapolation of log conditional Cu^{II} affinities
Extrapolation of log conditional Cu^{II} affinities of $K^{21}(Cl_2CF)$-Hepcidin determined in different percentages of acetonitrile (MOPS, pH 7.4). $y = -0.0058x + 7.22$

(A)

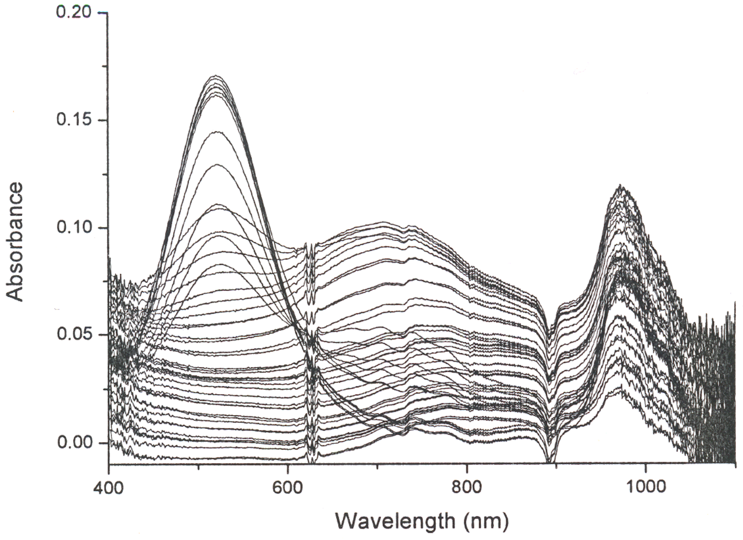

(B)

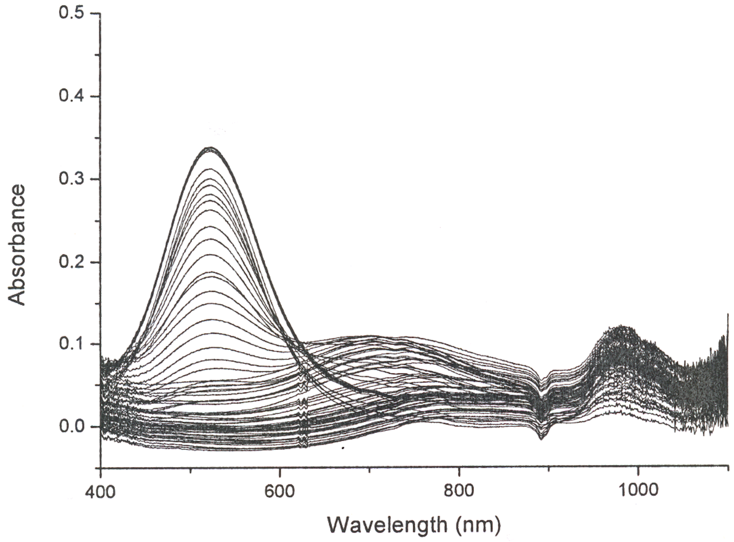

Figure S6 Spectrophotometric titration spectra of DTHFPIAIF-NH_2 with Cu^{II}
(A) Titration spectra of DTHFPIAIF-NH_2 with Cu^{II} in a acetonitrile:0.1M KCl solution (50:50). [DTHFPIAIF-NH_2] =1.18 mmol/L and [Cu^{II}]=0.29 mmol/L, ratio of L:M = 4.1, initially 15.28 mL of 0.1M KCl at 25° C, pH from 2.04 to 6.60.
(B) Titration spectra of DTHFPIAIF-NH_2 with Cu^{II} in a acetonitrile:0.1M KCl solution (1:2). [DTHFPIAIF-NH_2] =2.54 mmol/L and [Cu^{II}]=0.61 mmol/L, ratio of L:M = 4.2, initially 15.60 mL of 0.1M KCl at 25° C, pH from 1.82 to 6.69.

Table S1 Extrapolation of log conditional CuII affinities for DTHFPIAIF-NH_2

Percentage Acetonitrile in 0.1 M KCl solution	50	33	0[a]
$LogK_{7.4}$[b]	8.7	8.4	7.8
$pCu^{II}_{7.4}$[c]	9.7	9.4	8.8

[a] Extrapolation from the 50% and 33% stability constants

[b] log conditional Cu^{II} affinities at pH 7.4, recalculated from pH-independent stability constants

[c] $pCu^{II}_{7.4}$ under the conditions $[ligand]_{Total} = 10^{-5}$M, $[Cu^{II}]_{Total} = 10^{-6}$M, pH = 7.4

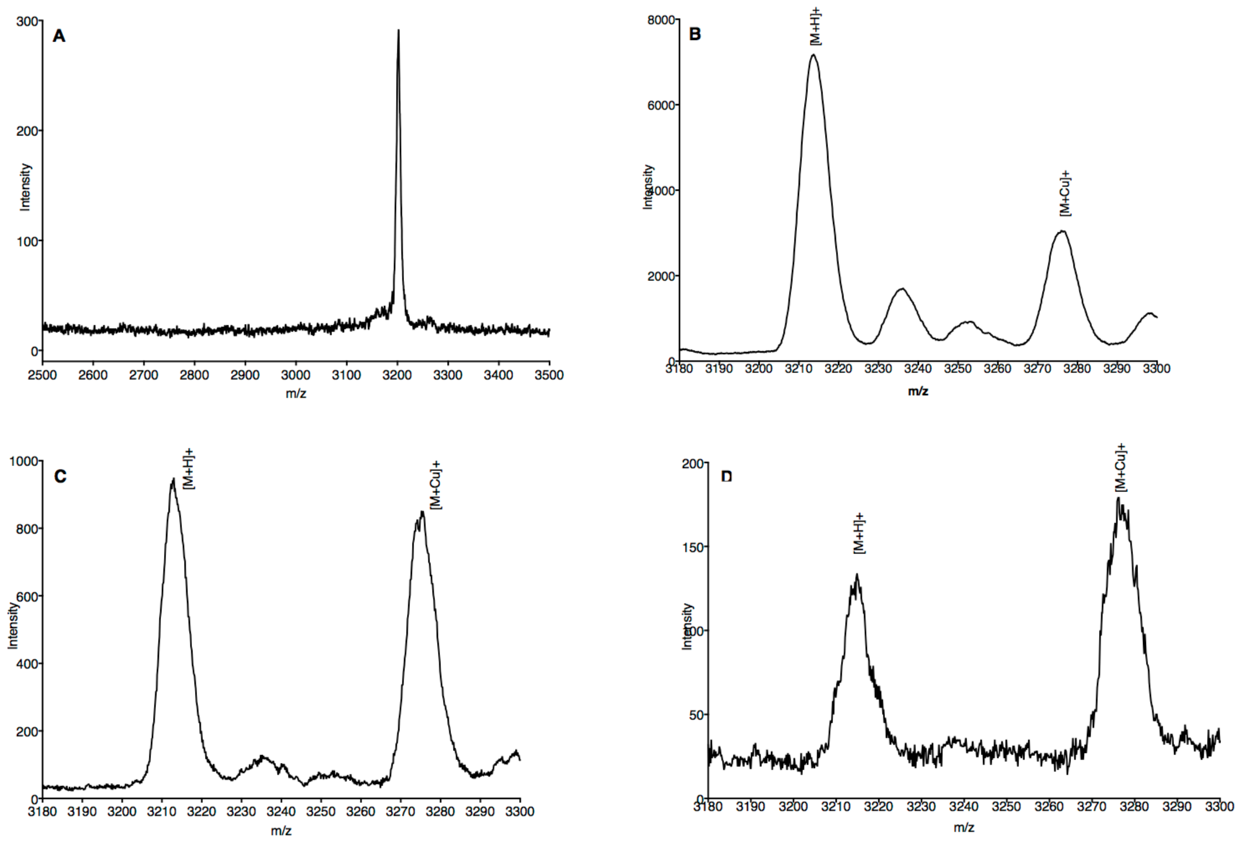

Figure S7. Analysis of Hepcidin-Cu^{II} complex using MALDI-TOF MS in the linear mode.

Hepcidin and Cu^{II} are present in equimolar amounts. (A), 2 nmol/L at pH 3; (B), 15.6 nmol/L at pH 7.0; (C), 500 nmol/L at pH 7.0; (D), 600 at pH 7.0 nmol/L

Ferroportin Internalizatio Assay

A stable cell line (Madin-Darby Canine Kidney, MDCK) was generated, which constitutively expressed human ferroportin (Fpn) fused at its C-terminus to the HaloTag protein (Promega Corp.). The internalization of Fpn was traced by labeling these cells with the fluorescent HaloTag-TMR (tetramethylrhodamine) ligand, which covalently attached to the HaloTag protein. Fpn-HaloTag MDCK cells were seeded in 100 µL DMEM medium (Dulbecco's Modified Eagle Medium with 10% fetal bovine serum (FBS) containing 1% Penicillin, 1% Streptomycin and 450 µg/ml G-418) per well of 96 well microplates (MicroClear, Greiner). After overnight incubation at 37°C/5% CO_2, the HaloTag-TMR ligand was added to the cells at 2 µmol/L final concentration and incubated for 15 min. at 37°C/5% CO_2. Cells were washed with Phosphate Buffered Saline (PBS), 100 µl DMEM medium was added and the cells were incubated for 30 min. at 37°C/5% CO_2. The medium was replaced with 50 µl fresh DMEM medium and 50 µl of the test compound was added diluted in DMEM medium. Hepcidin dose-response experiments were carried out with either 8 or 11 concentration dilution series of CF-hepcidin analogues and reference hepcidin (Bachem, Cat. No H-5926) spanning concentration ranges of 4 µmol/L to 0.2 nmol/L for labelled-hepcidin analogues and 2 µmol/L to 0.03 nmol/L for the reference hepcidin. Dose-response experiments were carried out with either 3 wells or 4 wells per peptide concentration for 8 and 11 concentration dilution series, respectively. After overnight incubation at 37°C/5% CO_2, 25 µL of Draq5 (Biostatus, Cat. No DR51000) was added to 2.5 µmol/L final concentration and incubated for 10 min. at 37°C/5% CO_2 to stain cell nuclei. After washing the cells with 200 µl of DMEM medium without phenol-red (GIBCO, Cat. No 11880), cells were fixed in 100 µl of 4% Paraformaldehyde (PFA, Electron Microscopy Sciences, Cat. No 15710-S) in PBS for 15 minutes at room temperature. The PFA solution was removed and cells were washed with PBS leaving 100 µl per well and plates were sealed with foil plate seal. TMR (520-550 nm excitation / 560-630 nm emission / 1000 ms

exposure time) and Draq5 (620-640 nm excitation / 650-760 nm emission / 200 ms exposure time) fluorescence images were acquired using the Operetta high content screening system (Perkin Elmer) using a 20x long working-distance objective. Four pictures were acquired per well and fluorescence channel covering ca. 2000 cells per well. The acquired image data was imported into the Columbus image data storage and analysis system (Perkin Elmer) for analysis. Image analysis included detection of nuclei (Draq5 fluorescence), definition of cytoplasmic region followed by application of the Ridge SER algorithm to analyze the texture of the TMR fluorescence in the cytoplasmic region as a quantitative measure for subcellular localization of ferroportin. High TMR SER Ridge values correlated with Fpn localized to the cell surface and low TMR SER Ridge values with the absence of Fpn from the cell surface. Relative values for Fpn internalization in percent were calculated as follows: Fpn Internalization (%) = (1 – ((SER Ridge – Average of SER Ridge of 1000 nmol/L hepcidin)/(Average of SER Ridge of 0 nmol/L hepcidin – Average of SER Ridge of 1000 nM hepcidin))) * 100. EC_{50} values were calculated with the relative Fpn internalization data using “log(agonist) vs. response” curve fitting of Prism 5 software (GraphPad Software Inc., version 5.02). For each data set the fit of the “log(agonist) vs. response (three parameters)” model was compared to the fit of the “log(agonist) vs. response – Variable slope (four parameters)” model and the EC_{50} data of the preferred model was used.
